# Supplementary material for: Lightning strikes as a major facilitator of prebiotic phosphorus reduction on early Earth
Source: Nat Commun. 2021 Mar 16;12:1535. doi: 10.1038/s41467-021-21849-2 (PMC7966383; doi:10.1038/s41467-021-21849-2)
Supplement: Supplementary file 1 — Supplementary Information [file 41467_2021_21849_MOESM1_ESM.pdf]

Supplementary Information

Lightning strikes as a major facilitator of prebiotic phosphorus reduction on early Earth

Benjamin L. Hess*, Sandra Piazolo, and Jason Harvey

*Corresponding author, email: benjamin.hess@yale.edu

Supplementary Figures

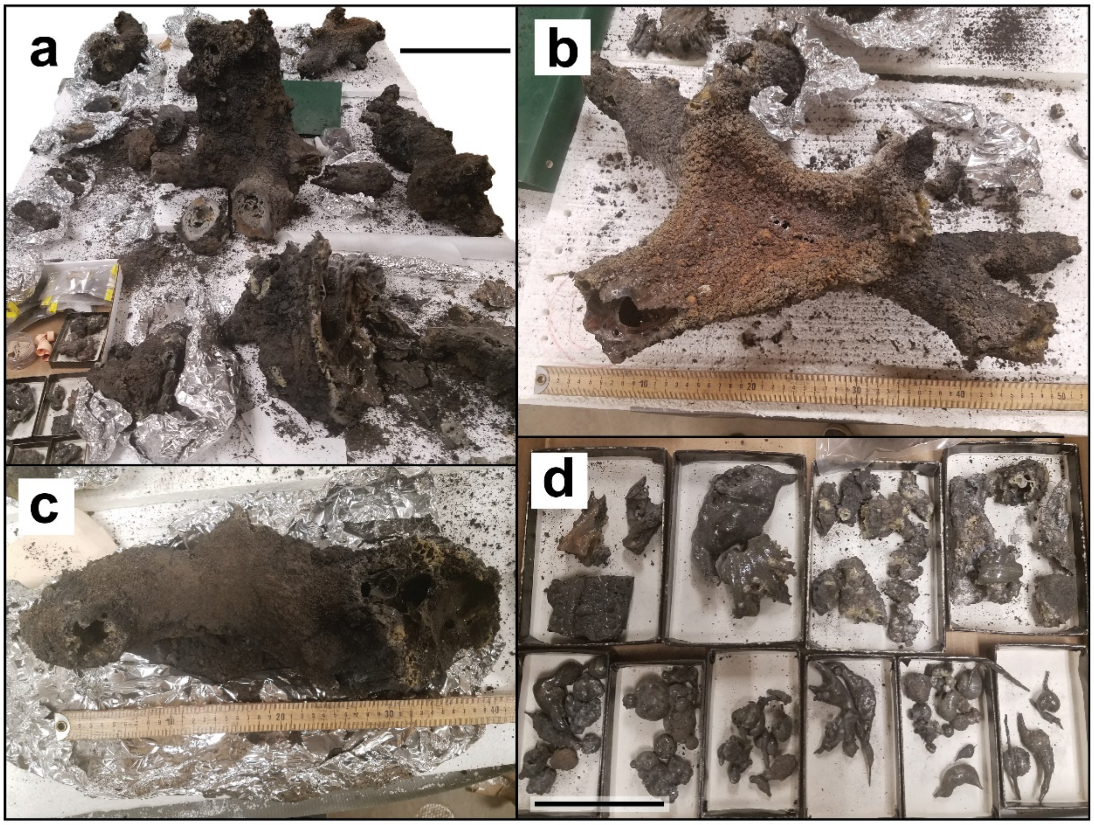

Supplementary Figure 1. Main pieces of the studied fulgurite. a) All major excavated pieces of the fulgurite weighing approximately 25 kg. The centre piece (see Fig. 1) was the main trunk of the fulgurite. The top of it was exposed at the surface prior to excavation. The base of the main trunk divides into several branches that intruded the soil parallel to the surface. Scale bar = 20 cm. b) and c) Two of the larger branches; metre stick for scale. (d) Glass droplets collected at the surface around the fulgurite site. Scale bar = 10 cm.

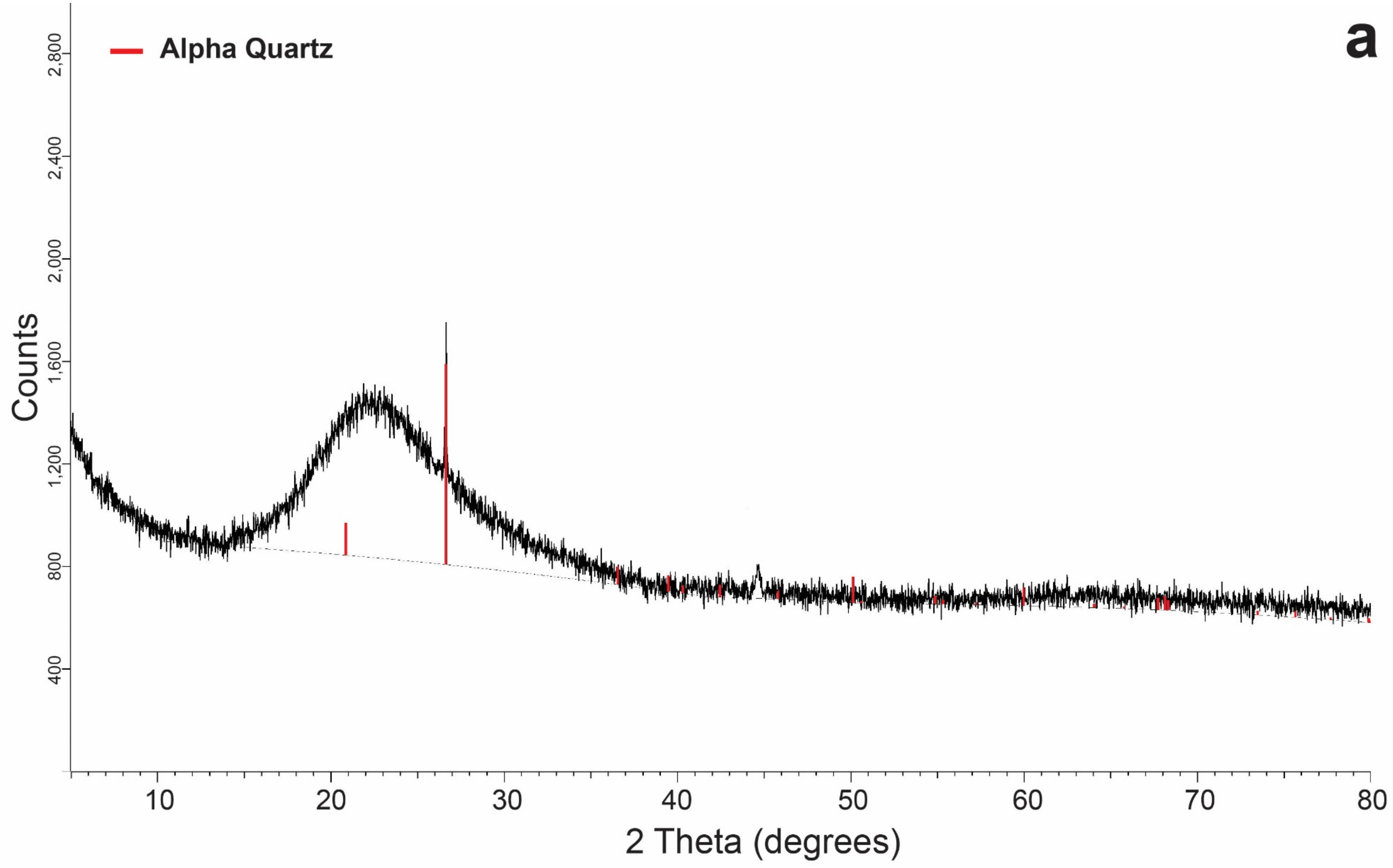

Alpha Quartz
a
Counts
2,800
2,400
2,000
1,600
1,200
800
400
10
20
30
40
50
60
70
80
2 Theta (degrees)

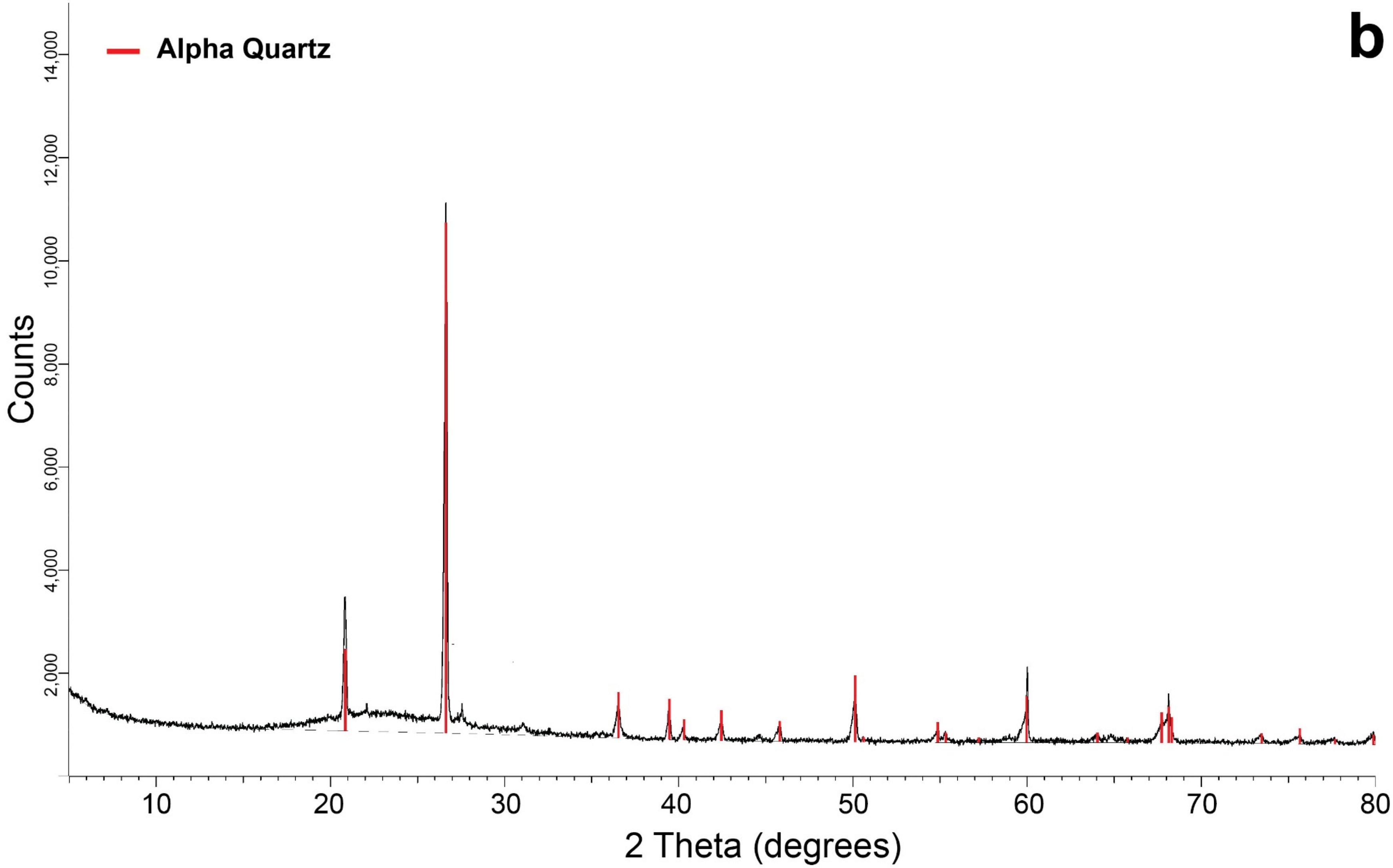

Alpha Quartz
b
Counts
14,000
12,000
10,000
8,000
6,000
4,000
2,000
10
20
30
40
50
60
70
80
2 Theta (degrees)

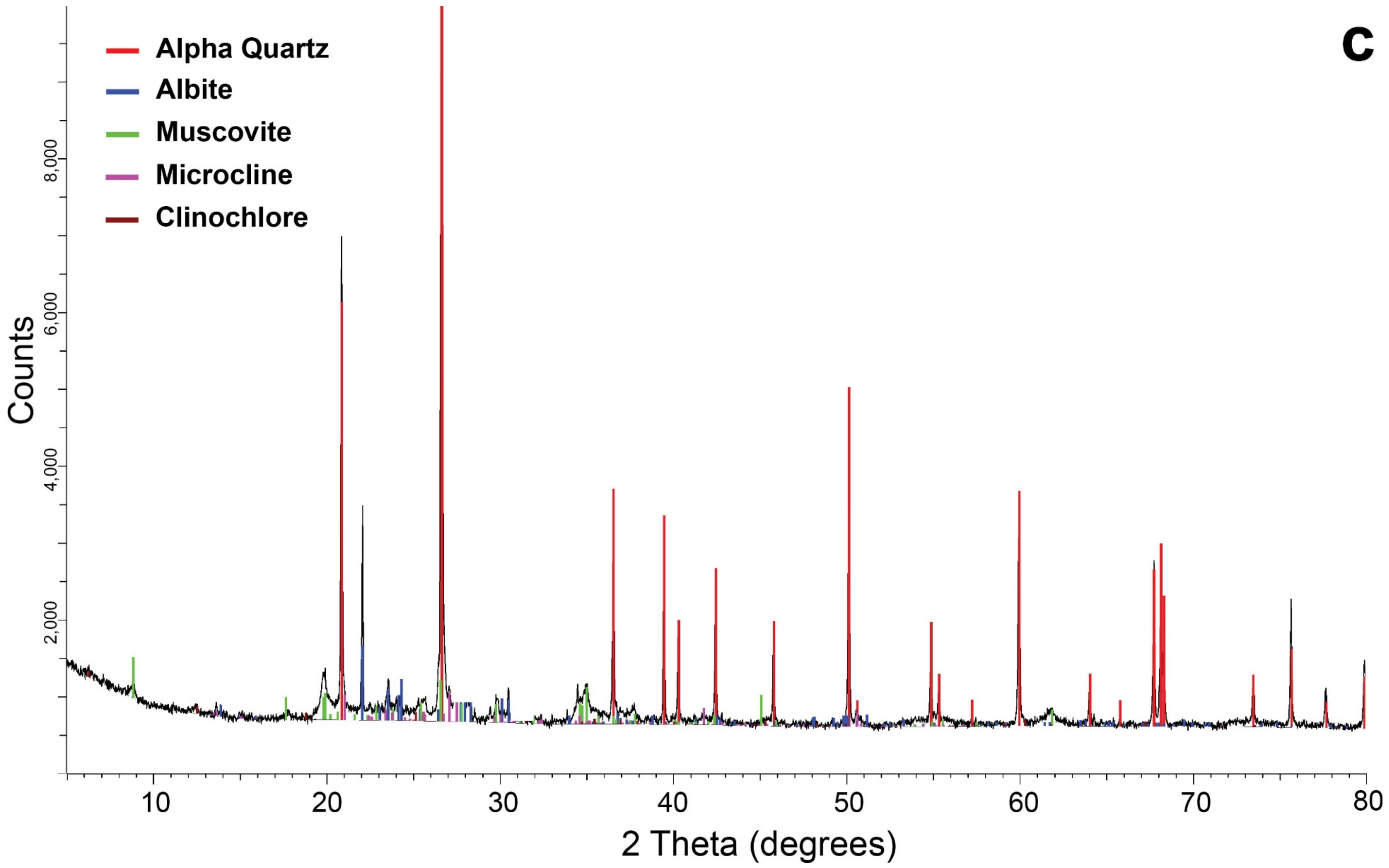

Supplementary Figure 2. X-ray Diffraction (XRD) results for the fulgurite core, rim, and parent soil. a) XRD results show that the core of the fulgurite is amorphous glass with hints of quartz peaks. b) The rim is less amorphous and contains clear quartz peaks. c) The parent soil contains quartz, albite, muscovite, microcline, and clinochlore.

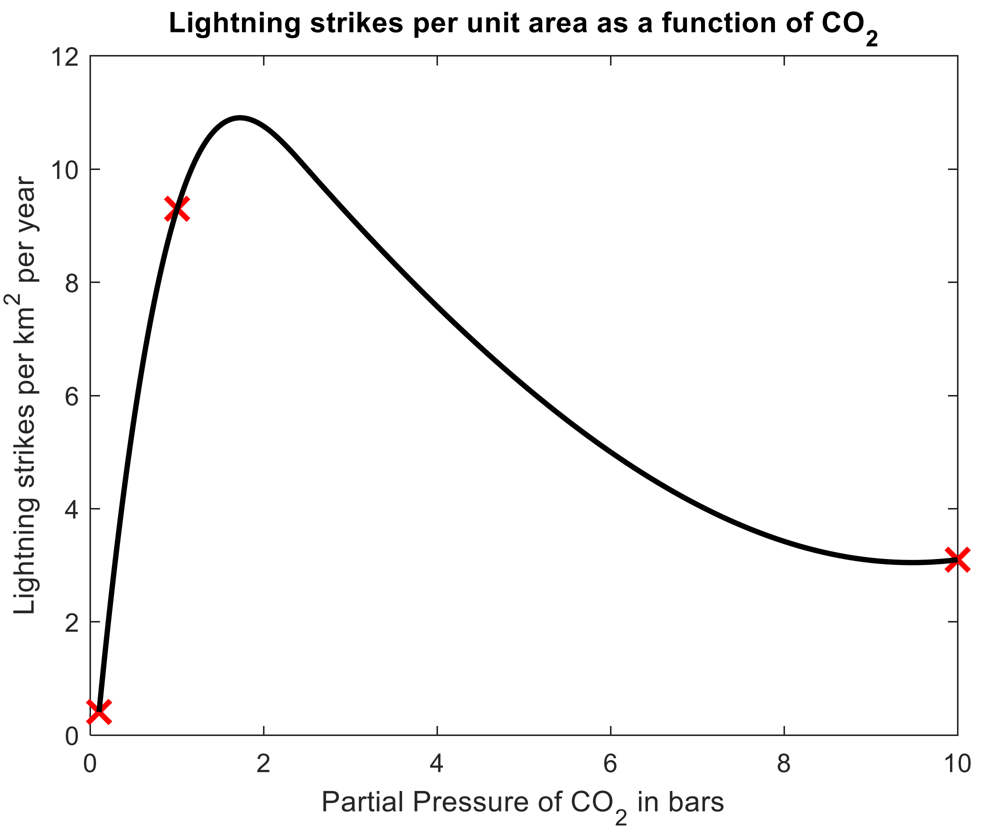

Supplementary Figure 3. Lightning strikes per km^2 per year as a function of pCO_2. Data points (marked with red 'x's) come from Wong et al.[1] and were fitted with cubic splines to make a smooth curve. See Supplementary Discussion 2 for further details.

Supplementary Tables:

Sample	Parent soil (wt %)	Uncertainty	Fulgurite rim (wt %)	Uncertainty	Fulgurite core (wt %)	Uncertainty	Measured Standard (wt %)	% Uncertainty	Certified Standard Value (wt %)
SiO_2	69.35	0.824	75.39	0.896	75.92	0.902	58.20	1.19	58.90
TiO_2	0.67	0.080	0.75	0.089	0.77	0.092	0.70	11.96	0.80
Al_2O_3	10.38	0.240	13.83	0.320	12.17	0.282	11.82	2.31	12.10
Fe_2O_3	4.13	0.092	4.31	0.096	4.63	0.103	5.57	2.22	5.70
MnO	0.15	0.005	0.16	0.005	0.16	0.005	0.19	3.17	0.20
MgO	0.99	0.055	1.07	0.059	1.09	0.061	2.22	5.56	2.10
CaO	0.96	0.001	1.22	0.001	0.98	0.001	4.00	0.08	4.00
Na_2O	0.75	0.061	0.86	0.069	0.85	0.068	2.92	8.04	2.70
K_2O	2.44	0.042	2.76	0.048	2.80	0.048	1.57	1.73	1.60
P_2O5	0.22	0.015	0.11	0.007	0.16	0.010	0.21	6.67	0.20
LOI	10.16		0.90		-0.51		11.60		11.60
Total	100.19		101.33		99.00		99.02		99.90

Supplementary Table 1. X-ray Fluorescence (XRF) results for the parent soil, fulgurite rim, and core. The negative loss on ignition (LOI) for the fulgurite core indicates weight gained by the oxidation of highly reduced phases such as Fe. A Canadian certified standard stream sediment (STSD4) with a similar composition to the fulgurite was measured along with the samples. The uncertainty is determined by calculating the percent difference between the certified value and the measured standard value. STSD4 was created as part of the Canadian Certified Reference Materials Project (CCRMP) by the company CANMET Mining and Minerals Sciences Laboratories.

Supplementary Discussions:

Supplementary Discussion 1

In Figure 3a all oxides except P_2O_5 fall on the equivalent composition line between the soil and fulgurite. This line is soil composition normalized to an anhydrous and organic-free composition using the loss on ignition (LOI) value (Supplementary Table 1) and represents the composition the fulgurite should have if it is the same as the parent soil. All oxides fall along this line except for P_2O_5. Given the homogeneity of all other oxides, it seems reasonable to assume phosphorus should be about the same as well. Therefore, we attribute the deviation to the abundant, yet heterogeneously distributed schreibersite (Fe_3P) throughout the fulgurite core and rim (Figs. 2d-f).

Consequently, the difference between the value of the equivalent composition line and the measured P_2O_5 in the fulgurite is the minimum amount of P_2O_5 that was depleted from the glass matrix analysed with XRF and concentrated in Fe_3P. The maximum value would be if all the expected P_2O_5 were converted to schreibersite.

To calculate the minimum Fe_3P formed:

1. We determine what the expected amount of P_2O_5 based on the equivalent composition line (Fig. 3a) using the LOI value from Supplementary Table 1

$$P_{\text{expected}} = \frac{P_{\text{soil}}}{1 - \text{LOI}} = \frac{0.22\%}{(1 - 0.106)} = 0.246\%$$

2. Convert the expected P_2O_5 and fulgurite P_2O_5 wt% into mols

$$P_{\text{expected}} = 0.00246 \frac{\text{g P}_2\text{O}_5}{\text{g fulgurite}} * \frac{\text{mol P}_2\text{O}_5}{142\text{ g P}_2\text{O}_5} * \frac{2\text{ mol P}}{\text{mol P}_2\text{O}_5} = 3.47\text{x}10^{-5} \frac{\text{mol P}}{\text{g fulgurite}}$$

$$P_{\text{rim}} = 0.0011 \frac{\text{g P}_2\text{O}_5}{\text{g rim}} * \frac{\text{mol P}_2\text{O}_5}{142\text{ g P}_2\text{O}_5} * \frac{2\text{ mol P}}{\text{mol P}_2\text{O}_5} = 1.55\text{x}10^{-5} \frac{\text{mol P}}{\text{g rim}}$$

$$P_{\text{core}} = 0.0016 \frac{\text{g P}_2\text{O}_5}{\text{g core}} * \frac{\text{mol P}_2\text{O}_5}{142\text{ g P}_2\text{O}_5} * \frac{2\text{ mol P}}{\text{mol P}_2\text{O}_5} = 2.25\text{x}10^{-5} \frac{\text{mol P}}{\text{g core}}$$

3. Calculate the difference between expected and measured P

$$P_{\text{expected}} - P_{\text{rim}} = 3.47\text{x}10^{-5} - 1.55\text{x}10^{-5} = 1.92\text{x}10^{-5} \frac{\text{mol P}}{\text{g rim}}$$

$$P_{\text{expected}} - P_{\text{core}} = 3.47\text{x}10^{-5} - 2.25\text{x}10^{-5} = 1.22\text{x}10^{-5} \frac{\text{mol P}}{\text{g core}}$$

4. We assume the difference between the expected and measured P values is entirely a result of schreibersite formation and noting that there is a 1:1 P:Fe_3P molar ratio, we calculate the wt% of Fe_3P formed.

$$\mathrm{Fe_3P\ (rim)} = 1.92\mathrm{x}10^{-5}\,\frac{\mathrm{mol\ Fe_3P}}{\mathrm{g\ soil}} * \frac{198.5\ \mathrm{g}}{\mathrm{mol\ Fe_3P}} = 0.0038\ \frac{\mathrm{g\ Fe_3P}}{\mathrm{g\ rim}}$$

$$\mathrm{Fe_3P\ (core)} = 1.22\mathrm{x}10^{-5}\,\frac{\mathrm{mol\ Fe_3P}}{\mathrm{g\ fulgurite}} * \frac{198.5\ \mathrm{g}}{\mathrm{mol\ Fe_3P}} = 0.0024\ \frac{\mathrm{g\ Fe_3P}}{\mathrm{g\ core}}$$

5. Using the fulgurite core as a lower limit, we multiply by approximate size of the entire fulgurite (25 kg) to determine total mass of Fe_3P.

$$\mathrm{Fe_3P\ (core)} = 0.0024\frac{\mathrm{g\ Fe_3P}}{\mathrm{g\ core}} * 25{,}000\ \mathrm{g} = 60\ \mathrm{g}$$

Thus, we predict a minimum of 60 g of Fe_3P was formed in the studied fulgurite. Additionally, a maximum value can be determined by assuming that the fulgurite P abundance should fall on the equivalent composition line (Fig. 3a) and converting it entirely from P_2O_5 to Fe_3P.

$$3.47x10^{-5}\frac{\mathrm{mol\ P}}{\mathrm{g\ fulgurite}} * \frac{\mathrm{mol\ Fe_3P}}{\mathrm{mol\ P}} * \frac{198.5\ \mathrm{g}}{\mathrm{mol\ Fe_3P}} = 0.0069\frac{\mathrm{g\ Fe_3P}}{\mathrm{g\ fulgurite}}$$

$$0.0069\frac{\mathrm{g\ Fe_3P}}{\mathrm{g}} * 25{,}000\ \mathrm{g} = \ 172.5\ \mathrm{g\ Fe_3P}$$

Thus, the range of Fe_3P formed in the fulgurite is calculated to be between 60 and 172.5 g.

Supplementary Discussion 2

Estimating the annual mass of reduced phosphorus generated from lightning strikes has two steps: (1) estimating the number of fulgurite-forming lightning strikes per year and (2) estimating the amount of phosphorus reduced in each fulgurite. Since the output of our model provides a range based on order of magnitude, the exact input values are less important so long as the order of magnitude is approximately correct.

To estimate the number of fulgurite-forming lightning strikes per year, we use results from Wong et al.[1], who use the generic LMDZ 3D global circulation model (GCM) to estimate average lightning rates on early Earth as a function of pCO_2 in the atmosphere. The rationale behind this is that pCO_2 is a major control on mean surface temperature which in turns controls storm frequency, intensity, and consequently lightning in the GCM[2]. Consequently, pCO_2 can be used to estimate the average global lightning frequency to first order. Thus, we must determine a pCO_2 function to use.

We develop a pCO_2 function from the discussion of Kasting[3], assuming (1) after the moon-forming impact, pCO_2 would have been extremely high (tens of bars)[4]. (2) This value would rapidly decline during the early Hadean as high pCO_2 would lead to rapid carbonate formation. (3) A minimum value of about 0.2 bars pCO_2 must be maintained throughout the Hadean and early Archean to prevent the oceans from freezing over[5], curtailing silicate weathering and carbonate formation and allowing volcanic outgassing to increase pCO_2[5]. pCO_2 levels at least this value is also supported by studies suggesting liquid water-driven weathering in the Hadean[6,7]. Thus, 0.2 bars pCO_2 is the minimum value. We model the pCO_2 asymptotically decreasing to 0.2 bars throughout the first 1 billion years of Earth history by fitting a logarithmic curve to two points: 10 bars at ~4.5 Ga, and 0.2 bars at 3.5 Ga. The resulting function for pCO_2 as a function of time is:

$$y = -0.473\ln(x) + 3.4667 \qquad \text{(S1)}$$

This function is displayed in the inset graph of Figure 5c.

Next, we use the results of Wong et al.[1] to convert the pCO_2 into global lightning rates. Their three model runs give the following results:

pCO_2 (bars)	Lightning flashes per km^2 per year
0.1	0.41
1	9.3
10	3.1

We fit cubic splines to this data to generate a smooth curve shown in Supplementary Figure 3. Arguably, it is problematic that the curve fit predicts higher values than Wong et al.[1] between 1 and 3 bars, but given that from 4.3 Ga onward the pCO_2 value is lower than 1 bar (Fig. 5c), the impact of this on our analysis is negligible. The curves used to create Supplementary Figure 3 are as follows:

$$y_1 = -1.23 + 17.10x - 7.60x^2 + 1.02x^3, \quad [3 \geq x > 0.1] \qquad \text{(S2)}$$
$$y_2 = 14.67 - 1.97x + 0.028x^2 + 0.0054x^3, \quad [x > 3]$$

Inserting time into equation (S1) and then inserting that value into equation (S2) yields the lightning rate as a function of time shown in the inset graph of Figure 5c.

With a global lightning rate, we must next estimate how many fulgurites are formed annually. The requirements for fulgurite formation are (1) the strike occurs over land and (2) it makes contact with the ground. First, we determine how many lightning strikes there are per year. Taking the output of equation (S2) and multiplying by the surface area of Earth (approximately 510,000,000 km^2) gives the number of global flashes per year. For modern lightning, 75-90% of flashes occur over land[8]. Since we cannot know the land area and distribution on early Earth, we opt to make a similar assumption that lightning will preferentially strike over land. We choose to conservatively estimate between 25-75% of lightning flashes will be over land. Second, approximately 25% of lightning flashes are cloud-to-ground strikes[9], i.e., fulgurite forming. We

assume the same was true on early Earth. Thus, the number of fulgurite-forming strikes per year is a matter of multiplying the output of equation (S2) by 510,000,000, 0.25-75, and 0.25.

Lastly, we estimate the amount of P reduced per lightning strike. First, we assume that the average rock mass effected by a lightning strike will be 250 grams. This estimate is based on Elmi et al.[10] who studied a granite rock fulgurite. They estimate that the fulgurite mass formed per lightning strike "ranges from a few hundred grams to about 30 kg." Given the high melting point, specific heat, and low conductivity of igneous minerals, it is likely that fulgurites developed in an igneous rock will fall on the low side of this range. In contrast, in substrates such as soil, fulgurites may reach masses of about 30 kg (Supplementary Fig. 1). Consequently, an approximate mass of ~250 grams is reasonable for a fulgurite generated from lightning striking a carbonate and clay-rich igneous rock. It should be noted that so long as the order of magnitude (10^{2-3}) is correct, our results do not change significantly.

Second, we assume the struck material either has a komatiitic composition (0.0065 wt% P) or basaltic composition (0.044 wt% P; Fig. 3b). Third, we assume 5-10% of strikes will highly reduce and 25-50% of strikes will mildly reduce the struck material. Finally, we assume that for a highly reduced fulgurite, 10-20% of the phosphorus will become phosphide, and for a mildly reduced fulgurite, 25-50% of the phosphorus will become phosphite or hypophosphite. The estimated ranges in Figure 5c result from multiplying the upper and lower bounds of these values.

Supplementary Discussion 3:

Here we discuss the fate of reduced phosphorus from a large impactor. Using a power law scaling in accordance with the asteroid belt, more than 99.9% of the terrestrially accumulated meteoritic mass was probably held in meteorites larger than 1 km in diameter[11,12]. Therefore, understanding the fate of large impactors is critical to determining how available phosphide will be on Earth's surface.

Variables that affect the fate of the impactor are the angle at which it strikes the Earth, the speed of the strike, and the compositions of the impactor and the impacted surface[13]. Such large meteorites that strike at an angle greater than 30° entirely melt and partially vaporise[13] which may result in the oxidation of any reduced phosphorus species. However, it has been suggested that impact plumes would precipitate schreibersite, covering early Earth's surface in small spherules available for prebiotic chemistry[14]. There is an argument for such a process in lunar impact plumes[15], but it is not clear that this would occur on Earth as the thermodynamics and kinetic factors in impact plumes are complex[16].

Schreibersite formation requires sufficiently reducing ambient redox conditions or enough of a reducing agent, such as graphite. A large impact would melt and vaporise the impacted material as well as the impactor, causing mixing[13,17]. In both the terrestrial and lunar cases, the fO_2 of the crustal rocks is high enough that schreibersite would not form thermodynamically without a reductant, meaning schreibersite is highly unlikely to crystallize out of a standard melt[15,18]. In impact plumes, however, lunar schreibersite formation is proposed to have formed through kinetic rather than thermodynamic processes[15]. Under lunar redox conditions, volatilized phosphorus would form P and P_2 gases which would react with iron and rapidly form

schreibersite and cool quickly due to the expansion cooling of the plume[15]. Supporting the hypothesis that kinetics rather than thermodynamics controls the formation of lunar schreibersite, when lunar samples containing schreibersite are heated past 875 °C, schreibersite oxidizes to whitlockite, the stable phase under lunar redox conditions[19].

In contrast, the fO_2 of early Earth's crust was likely at least 2-3 log units above lunar rocks[18,20], and consequently, P would volatilize and form oxidized gases such as PO, PO_2, P_2O_5, and P_4O_{10}[15]. Additionally, the impact plume would likely entrain large amounts of atmospheric and vaporised water, which serves to increase the oxidation state of iron, forming wüstite, magnetite, and hematite, and preventing schreibersite formation[21]. It would likely also further oxidise phosphorus gases. It therefore seems unlikely that this mechanism would allow for schreibersite formation under terrestrial conditions.

An alternative mechanism is the same one at work in fulgurites: localized reduction via graphitic carbon in the graphite-CO buffer. Thermodynamically, this requires at least a 1:1 graphite to phosphorus ratio at lunar redox conditions and likely more, given the higher redox state of the Earth's crust and the likely water and atmospheric entrainment. Further, an impact plume is so turbulent and massive that it is seems unlikely that sufficient local concentrations of graphite, phosphorus, and iron could be maintained for the reduction required to form schreibersite spherules. Enstatite chondrites, which are thought to dominate Earth's late accretion phase[12], contain less than half a weight percent carbon[22]. Consequently, it is unlikely that there is sufficient graphite to allow for any significant or widespread graphite-based reduction in an impact plume. Therefore, it remains uncertain whether impact plumes would precipitate schreibersite. More rigorous thermodynamic and kinetic modeling are required to support this idea.

In Figures 5b and 5c we assume the amount of reduced phosphorus that survives the impact and is available at the surface to be between 5% and 50%. This allows our conservative estimates of lightning-based phosphorus reduction to be compared with generous estimates for meteorites. However, the meteoritic supply of reduced phosphorus is plausibly on the low end this range.

Supplementary References:

1. Wong, M. L., Charnay, B. D., Gao, P. Yung, Y. L. & Russell, M. J. Nitrogen oxides in early Earth's atmosphere as electron acceptors for life's emergence. *Astrobiology* **17**, 975-983 (2017).
2. Romps, D. M., Seeley, J. T., Vollaro, D. & Molinari, J. Projected increase in lightning strikes in the United States due to global weather. *Science* **346,** 851-854 (2014).
3. Kasting, J. F. Atmospheric composition of Hadean-early Archean Earth: the importance of CO. *Geol. Soc. Am. Spec. Pap.* **504,** 19-28 (2014).
4. Zahnle, K. J. Earth's earliest atmosphere. *Elements* **2,** 217-222 (2006).
5. Kasting, J. F. Theoretical constraints on oxygen and carbon dioxide concentrations in the Precambrian atmosphere. *Precambrian Res.* **34,** 205-229 (1987).
6.Mojzsis, S. J., Harrison, T. M. & Pidgeon, R. T. Oxygen-isotope evidence from ancient zircons for liquid water at the Earth's surface 4,300 Myr ago. *Nature* **409,** 178-181 (2001).
7. Trail, D. et al. Constraints on Hadean zircon protoliths from oxygen isotopes, Ti-thermometry, and rare earth elements. *Geochem. Geophys. Geosyst.* **8,** 1-22 (2007).

8. Lay, E. H., Jacobson, A. R., Holzworth, R. H., Rodger, C. J. & Dowden, R. L. Local time variation in land/ocean lightning flash density as measured by the World Wide Lightning Location Network. *J. Geophys. Res.* **112,** D13111 (2007).
9. Boccippio, D. J., Cummins, K. L., Christian, H. J. & Goodman, S. J. Combined satellite- and surface-based estimations of the intracloud-cloud-to-ground lightning ratio over the continental United States. *Mon. Weather Rev.* **129,** 108-122 (2001).
10. Elmi, C., Chen, J., Goldsby, D. & Giere, R. Mineralogical and compositional features of rock fulgurites: a record of lightning effects on granite. Am. Mineral. **102**, 1470-1481 (2017).
11. Bottke, W. F. et al. The fossilized size distribution of the main asteroid belt. *Icarus* **175,** 111-140 (2005).
12. Mojzsis, S. J., Brasser, R., Kelly, N. M, Abramov, O. & Werner, S. C. Onset of giant planet migration before 4480 million years ago. *Astrophys. J.* **881,** 44 (2019).
13. Pierazzo, E. & Melosh, H. J. Hydrocode modeling of oblique impacts: the fate of the projectile. *Meteorit. Planet. Sci.* **35,** 117-130 (2000).
14. Collins, G. S., Melosh, H. J. & Osinski, G. R. The impact-cratering process. *Elements* **8,** 25-30 (2012).
15. Pasek, M. A. Phosphorus as a lunar volatile. *Icarus* **255,** 18-23 (2015).
16. Ishimaru, R., Senshu, H., Sugita, S. & Matsui, T. A hydrocode calculation coupled with reaction kinetics of carbon compounds within an impact vapor plume and its implications for cometary impacts on Galilean satellites. *Icarus* **210,** 411-423 (2010).
17. Pierazzo, E. & Melosh, H. J. Melt production in oblique impacts. *Icarus* **145,** 252-261 (2000).
18. Trail, D., Watson, E. B. & Tailby, N. D. The oxidation state of Hadean magmas and implications for early Earth's atmosphere. *Nature* **480,** 79-82 (2011).
19. Taylor, L. W., Misra, K. C. & Walker, B. M. Subsolidus reequilibration, grain growth, and compositional changes of native FeNi metal in lunar rocks. *Proc. Lunar Sci. Conf.* **7,** 837-856 (1976).
20. Armstrong, K. Frost, D. J., McCammon, C. A. Rubie, D. C. & Ballaran, T. B. Deep magma ocean formation set the oxidation state of Earth's mantle. *Science* **365,** 903-906 (2019).
21. Gerasimov, M. V., Dikov, Y. P., Yakovlev, O. I. & Wlotzka, F. Experimental investigation of the role of water in impact vaporization chemistry. *Deep-Sea Res. Pt. II* **49,** 995-1009 (2002).
22. Wasson, J. T. & Kallemeyn, G. W. Composition of chondrites. *Phil. Trans. R. Soc. Lond.* **325,** 535-544 (1988).
